# Progress in Obstetrics and Gynecology1The second series of Harrington Lectures delivered at the University of Buffalo, May 24, 25, 26, 1910.

**Published:** 1910-08

**Authors:** J. Clarence Webster

**Affiliations:** Chicago Ill.; Professor of Obstetrics and Gynecology in Rush Medical College, affiliated with the University of Chicago; 706 Reliance Building


### BUFFALO MEDICAL JOURNAL.

Vol. LXVI. AUGUST, 1910. No. 1

### ORIGINAL COMMUNICATIONS.

### Progress in Obstetrics and Gynecology[1]

By J. CLARENCE WEBSTER, M.D.
Chicago Ill.
Professor of Obstetrics and Gynecology in Rush Medical College, affiliated with the University of Chicago.

Lecture I.—Obstetrics—Part I.

THE most ancient writings on medical subjects belong to Egypt, though the fragments possessed by us give but an imperfect idea of the exact status of medical science and practice in the early periods of its history. The most important document is the Ebers papyrus, now in the University of Leipzig. Its date was about 1500 B. C. It deals with the construction of the body and diseases of various parts, including special maladies of women.

The most important Egyptian medical works were six volumes of the series known as the Hermetic Books. Though these do not now exist, their titles are known, and it is interesting to note that one was a treatise on diseases of women. They were the authoritative guides to the practitioners of the time, priest-physicians. According to the Herodotus there was a fairly well-defined specialism in Egypt, though as regards obstetric practice it is probable that it was entirely in the hands of midwives, the references to obstetric subjects in the Ebers papyrus being very scanty.

In ancient Greece the practice of medicine was for a long period in the hands of the priests. Only in the centuries immediately preceding the Christian era did others enter the field, some actually studying diseases of the body, others working at the anatomy of animals, while still others became experts in the subject of personal hygiene.

Obstetric cases were attended by midwives who, according to Plato, were accustomed to give medicines to hasten labor, to tie and cut the cord, and to induce abortions. The genuine writings of Hippocrates contain very scanty accounts of obstetric and

1. The second series of Harrington Lectures delivered at the University of Buffalo, May 24, 25, 26, 1910.

gynecologic subjects and it is believed that he had no special knowledge of them.

After the death of Hippocrates, Greek culture gradually declined and was transferred to the mouth of the Nile, where it was manifested in its greatest glory in the Alexandrian Colony. Unfortunately the written records of this period have been almost entirely destroyed so that we know little about medicine or any other branch of knowledge. There can, however, be little doubt that the Alexandrian schools were more advanced than any which had previously existed in the civilised world. In support of this it is merely necessary to note that abdominal section was performed for intestinal obstruction, that suture of the intestine was practiced when the gut was incised and that suppurating kidney was treated by incision and drainage.

The dissection of dead bodies was carefully carried out and what is more interesting vivisection was actually practiced in the human subject, criminals being dissected alive in order that the unaltered appearance of the various structures might be noted. Two of the most famous authorities were Erasistratus and Herophilus (about 300 B.C.). The former of these operated on the liver and spleen, while the latter, distinguished for his anatomical researches, wrote a treatise on obstetrics. It is interesting to note that Xenophon of Cos, a successor of Erasistratus, was the first to check hemorrhage from vessels by ligature.

During the decadence of Greece and the development of Alexandria, Rome contributed comparatively little to medical knowledge. Indeed, it is certain that some of the most noted practitioners in the Capital city were Greeks especially from Alexandria. The first distinguished Roman physician of whom we have some record was Celsus (B.C. 53 to A.D. 7). In his work "De Medicina" there are several parts dealing with gynecology and abdominal surgery, but very few references to obstetrics.

In the writings of Pliny (A.D. 23 to A.D. 79) there are accounts of abdominal Cesarean sections performed on women dying in advanced pregnancy or labor, and it is stated that the first Scipio Africanus, and Manilius, a distinguished general, were brought into the world in this manner. Indeed, it was an ancient statute of Numa Pompilius (715 to 673 B.C.) that this operation was obligatory when women died in advanced pregnancy.

In the second century the most outstanding name is that of Soranus. Though there has been considerable discussion as to the identity of this writer, there is good reason for believing that he was educated in Alexandria before settling in practice at Rome. In his work on "Diseases of Women" considerable attention is given to obstetrics, evidently written for the benefit of midwives.

He gives them sound advice, warning them to be temperate, trustworthy, not greedy nor superstitious, nor to induce abortion for sake of gain. They should keep their nails closely cut so as not to injure the patient. They were to be instructed in dietetics, materia medica and minor surgical manipulations. He did not think they should know much about anatomy, but should be able to turn in abnormal presentations. He discusses menstruation, fecundity, the methods of preventing conception, and of inducing abortion. Normal labor is described in detail, delivery in the obstetric chair being mentioned. Reference is made to removing the membranes and to the fumigation of the uterus.

Galen, a native of Pergamos, born A. D. 130, after a course of study in Alexandria and other cities, settled in Rome in the time of Marcus Aurelius and had a very distinguished career as practitioner, writer, and teacher. His work covered the entire field of medicine, obstetrics and diseases of women included, though from his authentic writings he does not seem to have given special attention to them.

Specialism in medicine undoubtedly characterised practice in Rome at this period, obstetrics being almost entirely in the hands of the midwives though Soranus states that frequently in complicated cases male physicians were consulted. It is interesting to note that the midwives, to some extent, practised gynecology. This seems also to have been the case in Athens, though it was illegal. The first recorded contributions to medical literature by women were made by early Greek midwives. Thus, there is a work on menstruation by one Sotira now existing in the library of Florence. Lais, Elephantis, Salpe, and Aspasia also wrote on gynecological subjects.

In Rome there seems to have been a class of women physicians (not midwives) who went through regular courses of study and were known as feminæ medicæ. That they gave special attention to gynecology is certain, but as to whether or not they competed with the midwives is not known.

As the Roman Empire declined politically, medicine also declined as a science and during the long period of the Dark Ages, no workers of importance are known to have left any records. The growing christian church was antagonistic to science and especially opposed the study of the human body. It is not surprising that original investigations should have come to an end in the christian world.

Oribasius (4th century), Aetius (6th century), Paulus Aegineta (4th to 7th century?) were the last important writers of this era, but their works are compilations and record few original observations. In the large encyclopedia of Aetius, who practised in Constantinople, there is a volume on obstetrics and gynecology, which gives an interesting account of the knowledge of these subjects at the time. He considers such subjects as the signs of pregnancy, diagnosis of fetal sex, care of pregnant women, signs of labor, difficult labor, and the like. He quotes largely from Soranus, Galen, and older authors and it is interesting that several of his chapters were written by the female physician, or midwife, Aspasia.

Paulus Aegineta, who worked in Greece and Alexandria, appears to have given special prominence to diseases of women and obstetrics and is generally regarded as the first great male specialist and teacher of these subjects. He had a great influence among the Arabians by whom he was referred to as "The Obstetrician."

After the destruction of Alexandria by the Arabs in 646 A.D., Greek influence in medicine very markedly waned. Bagdad, under the ambitious Arabian Caliphs, became the new seat of learning. Later, Cordova, in Spain, became an important educational center. Translations of medical works taken from the Alexandrian libraries formed the chief source of instruction of physicians, who gradually came to form an important class in the Mohammedan world.

Of the prominent Arabian authorities whose names have come down to us, few are associated with obstetrics and gynecology, owing to the laws which forbade men to examine the genital organs of women, gynecological practice being consequently limited to women. It is known, however, that various important obstetric instruments were devised by the Arabians, and Albucasis, of Cordova, in the eleventh century described a case of ectopic pregnancy in which the fetus escaped by a suppurative process through the abdominal wall,—the first definite record relating to this form of gestation found in ancient writings.

During the period of Arabian dominance, medicine in western Europe was entirely neglected. There were no important writers or teachers or indeed, any professional class. With the revival of learning in the fifteenth century, Latin translations of the old Greek medical authors began to appear in Italy, leading anew to the study of the long neglected science. One of the first new works on obstetrics was that issued by Eucharius Rhodion, of Rhodes, in 1532. It was translated into English in 1552 by Thomas Reynold, under the title "The Byrth of Mankind," otherwise named "Woman's Boke."

That the publication of this work met with a good deal of opposition is evident from the preface in which reference is made to those who

"think it is not meete nor fitting such matters to be entreated of so plainly in our mother and vulgar language, to the dishonour

(as they say) of womanhood and the derision of their own secrets, by the detection and discovery thereof, men it reading or hearing, shall be moved thereby, the more to abhore and loath the company of woman, every boy and knave reading them as openly as the tales of 'Robin Hood.'"

In 1566, Caspar Wolf made a compendium of obstetric writings collected from older writers. Ambrose Paré published a work on obstetrics in 1567, therein describing version. Israel Spach published an encyclopedia of obstetrics in 1597. As male physicians began to give more attention to these subjects they met with strong opposition from the midwives. That popular feeling was, in some parts of Europe, in favor of the latter class is shown by the record of a physician being burned alive in Hamburg in 1521 for practising obstetrics.

The following quotation from an English pamphlet of the sixteenth century is of interest in this connection:

The men have but lately come into fashion. In praise of Scotland and Ireland be it spoken, the women of these countries are still too modest to employ them. What is the consequence? Adulteries happen very seldom in these countries; and every farm swarms with strong, healthy, well-limbed children.

Dr. Willughby, practising in England in the latter part of the seventeenth century, whose daughter was a midwife, has left on record the following account, illustrating the state of obstetrical practice at the time:

In Middlesex, anno 1685, my daughter, with my assistance, delivered Sir Tennebs Evank's lady of a living daughter. All the morning my daughter was much troubled, and told me that she feared that ye birth would come by ye buttocks. About seven o'clock that night labour approached. At my daughter's request, unknown to the lady, I crept into the chamber upon my hands and knees, and returned, and it was not perceived by ye lady. My daughter followed me, and I being deceived through hast to go away, said that it was he head, but she affirmed the contrary. However, if it should prove ye buttocks, that she knew how to deliver her. Her husband's great Oliverian power, with some rash expressions that he uttered, flowing too unhandsomely from his mouth, dismayed my daughter. She could not be quieted until I crept privately again the second time into ye chamber, and then I found her words true. I willed her to bring down her foot, the which she soon did, but being much disquieted with fear of ensuing danger, shee prayed mee to carry on the rest of the work."

One of the strongest opponents of male obstetricians in Europe in the seventeenth century was Louyse Bourgeois, the most famous midwife of Paris. She was a strong-minded woman and expended a good deal of violent language in denouncing those who ventured to trespass in her sphere of work. Two physicians in particular were favored with her strongest invective—namely, Guillerneau, and Honoré. These attacks were made as early as 1600 and it is, therefore, clear that Astruc was in error when he wrote that males began to practise obstetrics in France about 1663.

Louyse Bourgeois had the patronage of the Court and carried on a very large practice in aristocratic circles. One of the infants delivered by her was Henrietta Maria, afterward wife of Charles I of England. She also had the honor of having been the first modern woman to write a work on midwifery, though the honor is of doubtful value, since it has been clearly shown that the book was to a great extent a resumé of Ambrose Paré's treatise of 1573. The lady made no acknowledgment of her indebtedness to this distinguished man and she achieved much popularity in the profession, her book appearing in several editions during the seventeenth century.

It was from the 1642 edition of Louyse Bourgeois's book that the first popular English textbook was chiefly compiled. This work continued to be a standard authority for many years. It is worthy of some notice as giving an account of the theory and practice of midwifery and diseases of women, as taught during the second half of the seventeenth century. The work was published in London in 1656 and was celled "The Compleat Midwife's Practice." It was edited by four physicians whose initials only are given on the title page. The editors state in the preface that the work gives the practice not only in English, but of Spanish, French, and other nationalities. They state their indebtedness to Louyse Bourgeois. On the title page are two biblical passages—namely, "Exod.1.17, But the Midwives feared God; v.20, Therefore God dealt well with the Midwives."

One great object in publishing the book was for the better teaching of midwives who, according to the editors, neglected "all the wholesome and profitable rules of art which might concern them in the occult diseases of women, as also the anatomical parts of the body." The first part of the book is concerned with midwifery, the second with diseases of women and children, the third with certain clinical discourses. There is an appendix in the form of a letter of instruction from a dying midwife to her daughter. At the beginning of the first part there is an interesting account of the anatomy and physiology of the male and female genitals. It is most interesting reading, and serves as a standard wherewith we can measure the advance made in our knowledge during the last two centuries.

In a chapter on the signs of conception the following sentence occurs: "Take the urine of a woman and shut it up for three days in a glass; if she have conceived at the end of three days there will appear in the urine certain live things to creep up and down." It is a pity that no account of these "live things" is given. The description must, of course, refer to the growth of colonies of microörganisms in the urine, and it is interesting to note that the appearance presented by standing urine was attributed to action of living organisms. It is not likely, however, that the microbial composition of the masses seen in the urine was known.

Two chapters deal with the diagnosis of the sex of the fetus during pregnancy. It is stated that when there is a male *in utero* "The woman's right eye will move swifter and look clearer than the left. The right pap will also rise and swell beyond the left. If you cast the milk of the woman upon the urine it will presently sink to the bottom. As the woman goes she always puts her right leg forward, and in rising she eases all she can her right side sooner than her left." As regards the conception of a female it is taught that "girls are begot of parents who are by nature more cold and moist, their seed being more cold and moist and liquid."

It was evidently part of the duty of midwives in the seventeenth century to teach pregnant women how to take care of themselves. A chapter of wholesome advice ends as follows: "As soon as ever they perceive themselves to be with child, they must lay aside their busks and not straighten (constrict) themselves in any way, for fear of hurting the fruit of their womb, by not giving it its full libertie of growth." To prevent the breasts from becoming too large several means were adopted, one of which was the wearing of a small gold or steel chain around the neck during pregnancy. To keep the abdomen firm and smooth, various ointments were used with or without a dressed dogskin. During the last month of confinement it was the rule to rub the belly, perineum, hips and thighs with ointment for the purpose of softening the parts.

Among the causes of parturition is mentioned: "the narrowness of the place where the infant lies, so that he is forced to seek room otherwhere, which makes him to break the membranes wherein he is contained, pressing and constraining the mother, by the sharpness of those waters, to do her duty for his release." As regards the lying-in, it is interesting to note that of all the methods of delivery employed "the best and safest is to lie in their beds," though it is particularly enjoined that the patient should not lie down too much during the first stage.

In the management of a case, the midwives are told particularly not to be too hasty. The ignorance of some women is referred to, who, in "their haste to be gone to other women, do tear the membranes with their nail, to the danger both of the woman and the child, which then remains dry," thus causing delay and increasing the pain. They are advised to keep their nails pared close and to wear no rings. "She ought to be courteous, sober, chaste, not repining, cholerick, or covetous; she ought to be prudent, wary and cunning, ofttimes to use faire and flattering words."

In the treatment of the third stage, among the many methods of assisting the delivery of the afterbirth is mentioned rubbing the belly with the hand; the reason assigned is that it causes a breaking of wind in the bowel, this condition having been considered an important cause of delay in the third stage. The value of this plan really consisted in its stimulation of the uterus. When this method failed it was taught that the hand should be introduced into the vagina for the purpose of removing the placenta.

(*Continued in September*).

706 Reliance Building.

